# PTPN11-Related Noonan Syndrome Predisposes to Multifocal Low-Grade CNS Tumors Harboring FGFR1 Variants

**DOI:** 10.21203/rs.3.rs-8662616/v1

**Published:** 2026-01-28

**Authors:** Gary Kohanbash, Scott Ryall, Sam E. Gary, Lindsey M. Hoffman, Robert Siddaway, Anne E. Bendel, Karen W. Gripp, Andrew W. Walter, Jordan R. Hansford, Amy A. Smith, Hong Wang, John M. Skaugen, Uri Tabori, Cynthia E. Hawkins, Alberto Broniscer

**Affiliations:** University of Pittsburgh; Hospital for Sick Children; University of Pittsburgh; Children’s Hospital Colorado; Hospital for Sick Children; Children’s Minnesota; Alfred I. duPont Hospital for Children; Alfred I. duPont Hospital for Children; Royal Children’s Hospital; Arnold Palmer Hospital for Children; University of Pittsburgh; University of Pittsburgh; Hospital for Sick Children; University of Toronto; Children’s Hospital of Pittsburgh

**Keywords:** Brain tumor, glioma, Noonan syndrome, PTPN11, FGFR1

## Abstract

**Purpose:**

To characterize the clinical, radiological, and molecular characteristics of CNS tumors associated with Noonan syndrome (NS) and other non-Neurofibromatosis type 1 RASopathies.

**Methods:**

Twenty-four patients with concern for NS underwent clinical and central radiological review in this multi-institutional study. Whole-exome sequencing, RNA sequencing, and methylation analyses of peripheral blood and/or tumor specimens were performed.

**Results:**

Nineteen (79%) of 24 participants had NS, 17/19 (89%) of which had a germline *PTPN11* variant; Nineteen of 24 participants (79%) were male. Seventeen (89%) patients with NS developed CNS cancers, including low-grade glioma, (LGG; pure pilocytic/pilomyxoid astrocytoma; *n*=9) and mixed dysembryoplastic neuroepithelial tumor (DNET; *n*=6). Five patients incidentally diagnosed did not undergo histological confirmation. Radiological review showed multifocal parenchymal tumors in 9 patients with NS, including histologically confirmed neoplasm (*n*=2), radiologic progression (*n*=6), or typical tumoral imaging (*n*=1). All LGGs in patients with NS and germline *PTPN11* variants except one (14/15; 93%) harbored somatic *FGFR1* abnormalities. RNA sequencing of 12 tumors detected *FGFR1* internal tandem duplication in one patient. Comparison with published data showed a statistically significant association between brain tumor occurrence and *PTPN11*-related NS, driven by two genotypes: NM_002834.5(PTPN11):c.182A>G (p.Asp61Gly) and c.417G>T (p.Glu139Asp). Ten patients with LGGs, including 7 (41%) with NS, required chemotherapy. After median follow-up of 7.5 years, one patient died of CNS cancer.

**Conclusion:**

*PTPN11*-related NS predisposes to multifocal pure and mixed LGGs confirmed by radiological, histological, and molecular characteristics. Targeting FGFR1-related pathways may provide new treatment approaches for patients with NS and LGGs.

## Introduction

Cancer predisposition syndromes are present in 8% to 21% of pediatric patients with central nervous system (CNS) cancers [1–3]. Inhibition of RAS signaling through germline abnormalities in *NF1*, as well as germline abnormalities in non-*NF1* RASopathies (NNFRAS), is associated with non-CNS cancers [4–6]. NNFRAS comprise a large and heterogenous group of disorders, the most common of which is Noonan syndrome (NS), with an incidence of up to 1:1,000 live births [7, 8]. Multiple case reports and one case series (n = 5 patients) have described the association of NNFRAS [5, 9–20], particularly NS, with low-grade glioma (LGG). However, no large-scale data exist that that characterize clinical, radiological, and molecular features driving tumorigenesis and patient outcomes [11, 14, 20, 21].

Based on the prevalence of NS, the overlap between germline abnormalities found in NNFRAS and somatic changes found in pediatric CNS cancers, and our institutional experience [7, 8, 22], we conducted this multi-institutional study consisting of the largest reported patient population to evaluate clinical, radiological, and molecular characteristics that underlie NS and development of LGG. Leveraging this large cohort, we unveil novel, consistent associations between NS and LGG, including multifocal involvement and co-occurrence of germline *PTPN11* and somatic *FGFR1* abnormalities, indicating potentially new FGFR1-targeted treatment options.

## Material and Methods

### Data and sample collection.

Once Institutional Review Board approval (University of Pittsburgh #19060202) was obtained at six participating institutions in the US and Australia, patients with NNFRAS and CNS cancers were recruited to this study. CNS cancers were either histologically confirmed or suspected based on imaging characteristics. Except for three deceased patients, informed consent to study participation was obtained from adult patients, parents, or legal guardians. Assent and re-consent at age of majority were obtained according to institutional standards. Clinical information consisting of demographics, characteristics of NNFRAS, diagnosis and therapy of CNS cancers, and survival were obtained from all research participants. An experienced pediatric neuro-oncologist (A.B.) performed central radiological review of pertinent scans, including MRI and CT at diagnosis and at most recent available follow-up.

### Whole Exome (WES) and RNA Sequencing.

Germline DNA was extracted from either blood or saliva, and DNA and RNA were extracted from tumor samples, mostly derived from formalin-fixed, paraffin-embedded (FFPE) tissue, following standard methods. WES was performed at Novogene Corporation Inc. (Sacramento, CA) or at UPMC Hillman Cancer Center Core Laboratory. For WES done at Novogene, extracted DNA was quantified using Qubit TM dsDNA HS Assay Kit (Thermo Fisher Scientific, Waltham, MA). DNA libraries were prepared using Agilent SureSelect system (Agilent Technologies, Santa Clara, CA) to capture DNA coding sequences. 400 ng of genomic DNA was processed through fragmentation, enzymatic end-repair and A-tailing, adapter ligation, RNA probe hybridization, and polymerase chain reaction (PCR) amplification. The quality of libraries was analyzed with the DNA NGS 3K Assay Kit and LabChip GX Chip (PerkinElmer, Waltham, MA). Libraries with an average size of 400 bp (range: 300–600 bp) were quantified by qPCR using the VAHTS Library Quantification Kit for Illumina (Vazyme, Nanjing, China). The libraries were normalized and pooled as per the manufacturer’s protocol. Sequencing was performed using the NovaSeq 6000 platform (Illumina, San Diego, CA) with 151 paired-end reads to an average target depth of 100X for germline and tumor samples. Methods used for WES at Hillman Cancer Center were previously published [23]. Hybridization of 100 ng of FFPE-derived RNA was performed using the Illumina TruSeq exome protocol following the manufacturer’s instructions. Sequencing followed methods previously described [23].

### Computational Analysis.

Raw exome sequencing reads were trimmed with Trimmomatic-v0.32 [24], aligned with bwa-mem-v0.7.8 [25], and processed with the GATK suite [26]. Somatic variants were called using the GATK HaplotypeCaller and Mutect2 [27], retaining those identified in both. Variants with coverage > 10X, a minimum variant allele frequency (VAF) > 0.05, which were predicted to be pathogenic in silico via PolyPhen2 [28], SIFT [29], and FATHMM were annotated using SnpEFF-v4.3k [30, 31]. Population filters were applied using GnomAD and variants arising in < 1% of the total population were retained [32]. Priority was assigned to the genes highlighted in **Supplemental Table 1** given their known associations with RASopathies and/or gliomas. Copy number variants (CNVs) were identified using both on- and off-target reads with CNVkit v0.8.6 [33]. Raw sequencing reads were processed with the GenPipes framework [34]. Reads were qualitatively trimmed with Trimmomatic-v0.32 and aligned to human genome build GRCh37-v75 using STAR-v2.5.0 [24, 35]. Fusion transcripts were identified using MetaFusion [36]. Results were filtered against an in-house database of fusions known to be clinically relevant and consisting of a minimum of three supporting split reads. Internal tandem duplications in FGFR1 were identified using CICERO [37].

### DNA Methylation Profiling.

DNA whole-genome bisulfite conversion was performed using the EZ DNA Methylation kit (Zymo Research). DNA from FFPE tissue was subsequently restored by using the Infinium FFPE DNA Restore kit (Illumina). Bisulfite-converted DNA was amplified, fragmented, and purified using the Infinium MethylationEPIC BeadChip Kit (Illumina) according to the manufacturer’s protocol, then hybridized to the BeadChip array (Illumina). The BeadChip array was washed, prepared, stained, and scanned on the Illumina NextSeq 550 (Illumina) per the manufacturer’s protocol. iDAT files were uploaded and classified using version 11b6 and 12.5 of the CNS tumor methylation classifier (https://www.molecularneuropathology.org/mnp/). A calibrated score cutoff of 0.9 was used to determine a successful diagnosis. Copy number variation (CNV) profiles were inferred using the R “conumee” package (http://bioconductor.org/packages/conumee/) as implemented in the classifier package.

### Statistical analysis.

Analysis of correlation between genotype and occurrence of brain tumors in patients with NS was performed by comparing patients’ germline findings in the current study with previously published data using its 95% confidence interval (CI) [38].

## Results

### Patient Demographics and Clinical Outcomes

Twenty-four patients were enrolled in this study, 19 (79%) of which were male. Twenty-two patients were younger than 18 years when diagnosed with CNS cancer. Clinical diagnoses consisted of NS (*n* = 19; 79%), cardiofaciocutaneous (*n* = 2), Legius, linear nevus sebaceous (LNS), and NS with multiple lentigines syndrome in one patient each (**Table 1**). All patients had overt phenotypes associated with NNFRAS and were diagnosed before identification of CNS cancers. Five patients without symptoms attributable to CNS tumors were incidentally diagnosed with tumors during work-up for initiation of growth hormone supplementation (*n* = 2), seizures (*n* = 2, both with infra-tentorial tumors), or developmental delay (*n* = 1). After a median follow-up of 7.5 years (range: 0.5–20.2+), one patient with a low-grade glioma died of tumor progression and 3 patients died of causes unrelated to tumor. Ten patients with LGGs received chemotherapy and/or targeted therapy and one with a high-grade glioma underwent local radiation therapy and chemotherapy. None of the patients with histologically confirmed or presumptive DNETs required anticancer therapy (**Table 1**).

### Germline Genetic Characteristics

**Figure 1:**
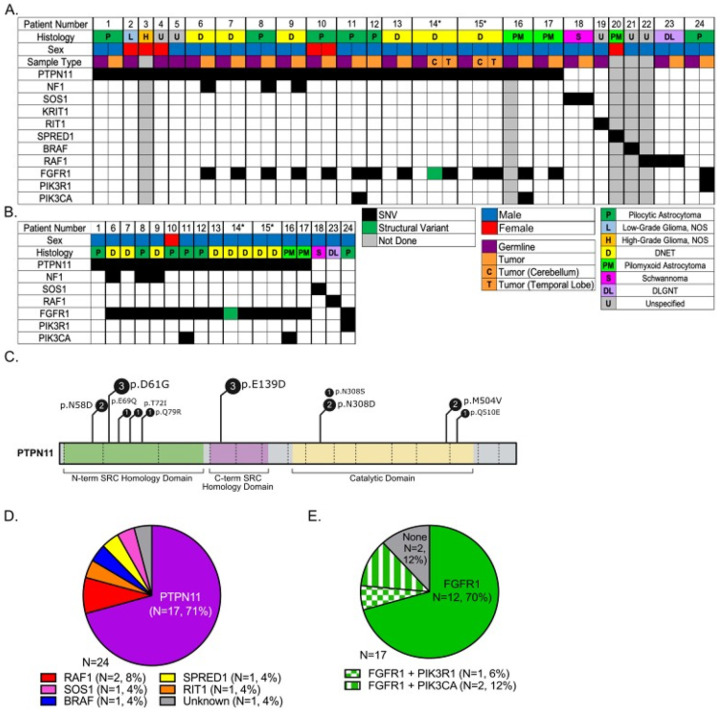
Molecular characterization of *PTPN11*-Related Noonan Syndrome Predisposed Multifocal Low-Grade Gliomas. (A) Oncoprint for all 24 patient. (B) Oncoprint for patients whose tumors underwent whole-exome sequencing. (C) Distribution of *PTPN11* germline variants. (D) Distribution of RASopathy driver mutations. (E) Secondary tumor molecular abnormalities.

Molecular germline analysis via WES of peripheral blood confirmed the diagnoses in 23 (96%) cases (Fig. 1A) and was used to compare to WES performed from tumor tissue (Fig. 1B). Germline pathogenic, gain-of-function *PTPN11* missense variants occurred in 17 (89%; 95% CI: 0.67–0.99) patients with NS (**Table 1**, Fig. 1A). The distribution of germline variants in *PTPN11* is shown in Fig. 1C. Two *PTPN11* genotypes, NM_002834.5(*PTPN11*):c.182A > G (p.Asp61Gly) and c.417G > T (p.Glu139Asp) were significantly overrepresented in our patients at a two-sided 0.05 significance level (**Supplemental Table 2**). The second most common NNFRAS driver mutation was *RAF1* (n = 2, [Figure 1D]). Interestingly, 12 (71%) patients with NS had germline *FGFR1* genetic abnormalities, including two with *FGFR1* and *PIK3CA* abnormalities and one with *FGFR1* and *PIK3R1* abnormalities (Fig. 1E).

### Genetic and Transcriptomic Characterization of Tumor Specimens

To better understand the genetic alterations associated with NS and CNS cancers, we performed WES from FFPE tumor specimens. WES was completed in 15 tumors from 13 (81%) of 16 patients with *PTPN11*-associated NS and a confirmed or suspected LGG, including paired multifocal tumors in 2 patients. Fourteen (93%) of 15 tumors harbored a somatic heterozygous *FGFR1* hotspot mutation or an internal tandem duplication (ITD) of its tyrosine kinase domain (*n* = 1) (Fig. 1B). While the cerebellar DNET of patient 14 harbored an *FGFR1* ITD, his temporal DNET had an NM_023110.3(FGFR1):c.1966A > G (p.Lys656Glu). Patient 15 had an NM_023110.3(FGFR1):c.1638C > A (p.Asn546Lys) in his cerebellar and temporal DNETs, respectively.

To identify splicing events and other alterations at the transcript level, RNA sequencing was performed on 12 tumors collected from 10 patients. An *FGFR1* ITD was detected in one of patient 14’s tumors. No other transcript fusions were detected.

### Epigenetic Characterization of Tumor Specimens

Whole-genome bisulfite sequencing was performed on 15 tumors, leading to changes in diagnosis for two patients. Patient 3 likely had a diffuse midline glioma, H3 K27M-mutant with *CDK4* and *MDM2* amplification (calibrated score of 0.87). Despite histological diagnosis of pilocytic astrocytoma, DNA methylation clustered patient 24’s tumor with rosette-forming glioneuronal tumors (calibrated score of 0.99). A summary of DNA methylation results is provided in **Supplemental Table 3**.

### Central Radiological Review and Histological Characteristics

**Figure 2:**
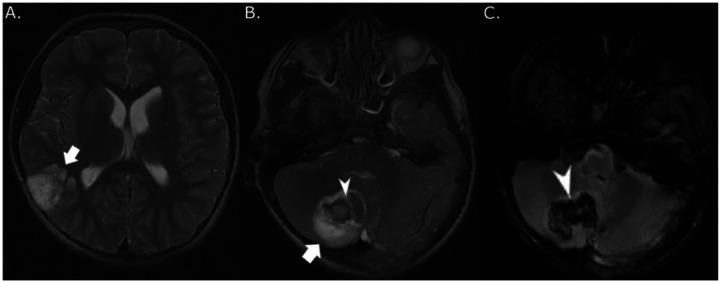
Radiologic characteristics of a multifocal DNET. (A) Axial T_2_-weighted MRI in patient 14 showed hyperintense, septated lesion in right temporal lobe (white arrow), which was confirmed to be a dysembryoplastic neuroepithelial tumor (WHO grade 1). The deeper aspect of the tumor was shown to have a hemorrhagic area by gradient echo sequence. (B) Axial T_2_-weighted MRI in patient 14 obtained on the same day showed a separate lesion in the right cerebellum (white arrow) with hyperintense rim and an eccentric isointense central area (white arrowhead) compatible with hemorrhage. This lesion was also confirmed to be a dysembryoplastic neuroepithelial tumor (WHO grade 1). (C) Gradient echo sequence confirmed cerebellar tumor hemorrhage (white arrowhead).

Detailed radiological data for all cases are provided in **Supplemental Table 4**. Primary tumor site was in the infra-tentorial space (*n* = 9), cerebral cortex (*n* = 8), diencephalon (*n* = 3), optic pathway (*n* = 2), and spinal cord (*n* = 1). Patient 14 had two separate synchronous tumors of equal size in the temporal lobe and cerebellum at diagnosis (Fig. 2) [16]. Nineteen patients underwent histological tumor confirmation, and five of six asymptomatic cases were diagnosed based on imaging only.

**Figure 3:**
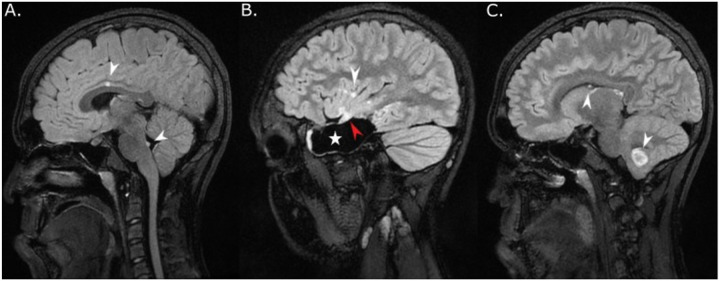
MRI of patient 9 with multifocal DNET and leptomeningeal dissemination. (A) Sagittal FLAIR post contrast image showed enlarging enhancing nodules in the dorsal brainstem (lower white arrowhead) and in the inter-hemispheric fissure (upper white arrowhead) compatible with progressive leptomeningeal disease 7 years after diagnosis of multifocal dysembryoplastic neuroepithelial tumor (WHO grade 1) with primary tumor in the right temporal lobe. (B) Sagittal FLAIR post-contrast image obtained on the same day showed residual hyperintense right temporal lobe tumor (red arrowhead) and surgical cavity (white star). Additional enhancing leptomeningeal punctate lesion was also seen in the right frontal lobe (white arrowhead). (C) Sagittal FLAIR post contrast image obtained on the same day showed a second distinct lesion in the right cerebellum with a typical ring sign (lower white arrowhead) and intra-ventricular punctate enhancing lesion compatible with leptomeningeal spread (upper white arrowhead).

Twelve (52%) of 23 evaluable patients, including 9 (47%) with NS, displayed multiple T_2_/fluid-attenuated inversion recovery (FLAIR) hyperintense, non-infiltrative, mostly non-enhancing and cystic, separate lesions deep in brain parenchyma without mass effect (Fig. 2 **and Supplemental Table 4**). Institutional radiological review had already prospectively detected multiple brain lesions in at least 9 cases. Two patients with NS (patients 14 and 15) underwent resection of two separate synchronous and metachronous tumors, respectively, in the temporal lobe and cerebellum (**Supplemental Table 4**). Among 10 patients without histological confirmation of distant parenchymatous lesions, a multifocal neoplastic process was confirmed based on slow radiological progression over a median of 6.6 years (range, 2.5 + to 20.2+) in 8 patients with NNFRAS, 6 of them with NS (**Supplemental Table 4**). Two patients had non-progressive multifocal distant tumors suspicious for a neoplastic process since they either shared imaging characteristics with the primary site (FLAIR ring sign [patient 9, Fig. 3]) or displayed mass effect with elevated choline and decreased N-acetylaspartate (NAA) on MR spectroscopy (patient 21)[39]. Multifocal brain lesions affected the posterior fossa (cerebellum and/or brainstem) and thalamus in 9 (100%) and 5 (56%) patients with NS, respectively, which are unusual locations considering their confirmed (*n* = 5) or presumptive (*n* = 2) diagnosis of dysembryoplastic neuroepithelial tumor (DNET). Five patients experienced intra-tumoral hemorrhage with or without intra-ventricular spread before diagnosis, including both patients with multifocal, histologically confirmed tumors (**Supplemental Table 4**). Excluding one participant with a diffuse leptomeningeal glioneuronal tumor (DLGNT), which almost uniformly presents with metastatic disease [10], five patients developed leptomeningeal tumor spread at follow-up confirmed radiologically only, including patient 9 who had a DNET and slowly progressive arachnoid disease (Fig. 3).

According to institutional histological review, diagnoses were pilocytic/pilomyxoid astrocytoma (WHO grade 1; *n* = 9 [47%]), DNET (WHO grade 1; *n* = 6 [32%]), and one each of LGG not otherwise specified (NOS; WHO grade 1), high-grade glioma NOS, schwannoma (WHO grade 1), and DLGNT (Fig. 1A). DNETs were diagnosed in both patients with histologically confirmed multifocal tumors. Central histological review of these 19 cases was not performed.

## Discussion

Our work provides the first large-scale clinical, radiological, and molecular study evaluating NS-associated brain neoplasms, which were mostly LGGs. There was an overwhelming male predominance observed in NS (79%), similar to previously published case reports (61%; **Supplemental Table 5**) [5, 9–20], for which this sex bias remains unknown. Brain tumors developed during childhood in most patients with NS and their continued slow growth during adulthood was documented in several cases. Further work is warranted to determine the behavior and repercussions of NS-associated brain tumors in adults.

The prevalence of germline *PTPN11* variants in NS is approximately 45% [40]. Herein, we demonstrated an overrepresentation of germline *PTPN11* and a genotype-phenotype association between NM_002834.5(PTPN11):c.182A > G (p.Asp61Gly) or c.417G > T (p.Glu139Asp) and the development of brain tumors in patients with NS. These *PTPN11* variants, as well as others, are known to induce protein changes and activate mitogen-activated pathway kinase (MAPK) signaling [38]. Additionally, we discovered a 93% co-occurrence rate of somatic *FGFR1* abnormalities and *PTPN11* germline variants in patients with NS and LGG. A previously reported *in vitro* cooperative effect of abnormalities in both genes was shown to cause overexpression of pERK compared to *PTPN11* variants alone in murine fibroblasts and was implicated in the tumorigenesis of pilocytic astrocytomas [41]. Similarly, a recent analysis of FGFR alterations across gliomas, including pediatric gliomas, support the hypothesis of a synergistic effect of PTPN11 and FGFR1 in the genesis of gliomas [42]. We propose that cooperative interaction between *PTPN11* and *FGFR1* abnormalities is critical for the tumorigenesis of LGGs in patients with NS, including DNETs. This has potential practical implications for patients with NS, as FGFR1 inhibitors have shown preliminary activity in LGGs harboring *FGFR1* variants [43]. Likewise, other inhibitors of the MAPK pathway (e.g., MEK inhibitors) may represent alternative therapies for patients with NS and LGG.

Multiple case reports described the presence of separate parenchymal lesions in patients with NS and NNFRAS [9, 17, 19], but only a few patients underwent histological confirmation [16, 44, 45]. Here, we show that multifocal parenchymal lesions in NS unequivocally represent synchronous or metachronous LGGs based on histological confirmation (*n* = 2; 22%), slow growth documented over a median follow-up of 6.6 years (*n* = 6; 67%), or typical radiological characteristics (*n* = 1, 11%). The only patient with NS without histological confirmation or radiological disease progression harbored a synchronous satellite lesion located in the cerebellum with FLAIR hyperintense ring sign, similar to the primary tumor, which is suggestive of DNETs [39]. Although this case was previously reported, no details about radiological findings had been provided [46]. The prevalence of multifocal tumors in 9 (53%) of 17 patients with NS and LGGs, a rate at least twice higher than those for patients with NF1 based on imaging [47], is striking and warrants confirmation in larger studies. Unlike NF1, only two (11%) of our patients with NS had primary involvement of the optic pathway [47], which further corroborates the distinct biological, clinical, and radiological tumor characteristics in patients with NS.

DNETs and other low-grade glioneuronal tumors accounted for approximately 50% of all CNS tumors in patients with NS and other NNFRAS reported to date [5, 10, 13, 14, 17–20, 45, 46, 48] (**Supplemental Table 5**). Likewise, DNETs were overrepresented and commonly displayed multifocal involvement in our patients, an otherwise rare phenomenon [49]. Therefore, the diagnosis of NS or other NNFRAS should be investigated in patients suspected to harbor multifocal LGGs. Based on a previous report [44], it is possible that individuals with NS and other NNFRAS could have histologically distinct multifocal LGGs. We also report a few unique cases, including the first association of Legius syndrome with a CNS cancer and a second case of a presumed brain tumor in a child with cardiofaciocutaneous syndrome. Unfortunately, no tumor tissue was available in either patient for molecular analysis.

Although most patients did not require anticancer therapy beyond surgery, 7 (41%) of 17 patients with NS and a confirmed or presumptive LGG required standard chemotherapy and/or targeted agents, 5 of whom %) received at least 2 different regimens. Overall, NS-associated CNS tumors are mostly non-life-threatening LGGs with indolent growth, but may present with complications, including disease progression, leptomeningeal spread, significant morbidity, and even death despite aggressive therapy.

One limitation of this study is its mostly retrospective design. Although 7 patients had been included in other reports [9–11, 16, 45, 46], we generated extensive new clinical, radiological, and molecular data about them. Based on the patients’ overt presentation of NS and NNFRAS, our findings may not be applicable to those with subtle features who are diagnosed based on molecular screening after the diagnosis of CNS tumors [17].

In conclusion, patients with NS and germline *PTPN11* variants account for nearly all individuals with NNFRAS and brain tumors in this study. Although approximately half of these patients harbored multifocal LGGs, their prognosis was good. However, anticancer therapy is required in a substantial minority of patients, particularly those with DNETs. Our finding that 93% of patients with NS and LGG harbor *FGFR1* mutations provides an exciting new therapeutic opportunity for these patients. Further investigation with CNS imaging is recommended for patients with NNFRAS presenting with neurologic signs and/or symptoms. Screening of asymptomatic patients with NM_002834.5(PTPN11):c.182A > G (p.Asp61Gly) or c.417G > T (p.Glu139Asp) variants for brain tumors may also be considered.

## Supplementary Material

Supplementary Files

This is a list of supplementary files associated with this preprint. Click to download.

- Table1.docx
- SupplTable1ListGenes.xlsx
- SupplTable2GenotypeDistribution.xlsx
- SupplTable3DNAMethylation.xlsx
- SupplTable4Radiology.xlsx
- SupplTable5PastExperience.xlsx
- SupplementalTableLegends.docx

## Data Availability

Deidentified whole exome sequencing, RNA sequencing, and DNA methylation sequencing data will be deposited to GEO or EGA and made freely available.

## Key points

- Multifocal low-grade glial and glioneuronal tumors are common in patients with Noonan syndrome, especially in males.
- Concurrent *PTPN11* and *FGFR1* variants exist in nearly all patients with Noonan syndrome and LGG.
- Targeting FGFR1may provide novel treatment options for patients with Noonan syndrome and LGG.

## Translational Relevance:

- Noonan syndrome (NS) has been recognized as a genetic predisposition to CNS tumors. However, no long-term clinical, radiological, or molecular patient data have previously been reported in a large patient cohort. Herein, we show that individuals with NS and germline *PTPN11* variants, particularly those with NM_002834.5(PTPN11):c.182A>G (p.Asp61Gly) and NM_002834.5(PTPN11):c.417G>C (p.Glu139Asp), are predisposed to develop low-grade gliomas (LGGs; pure and mixed glioneuronal), which may have multifocal distant brain involvement in up to 50% of patients. An overwhelming male predominance was confirmed, for which the biological underpinnings remain unknown. Somatic *FGFR1* variants were detected in 93% of LGGs in individuals with *PTPN11*-related NS. Therefore, concurrent variants in both genes seem to be required to drive gliomagenesis. Most LGGs experienced indolent behavior, even in the presence of residual disease, and the affected patients experienced long-term survival. However, aggressive monitoring and treatment may be necessary in a subset of patients.
